# Rural-origin health professional students’ perceptions of a support programme offered by Umthombo Youth Development Foundation

**DOI:** 10.4102/phcfm.v9i1.1212

**Published:** 2017-07-27

**Authors:** Dumisani M. Gumede, Andrew J. Ross, Laura M. Campbell, Richard G. MacGregor

**Affiliations:** 1 Umthombo Youth Development Foundation, South Africa; 2 Department of Family Medicine, University of KwaZulu-Natal, South Africa

## Abstract

**Background:**

Staffing of rural healthcare facilities is a challenge, with literature supporting the selection and training of rural-origin students. The Umthombo Youth Development Foundation (UYDF) scholarship scheme supports rural students to train as healthcare professionals and offers a unique support programme. This programme has not been evaluated, and this study sought UYDF-supported students’ perceptions of the programme.

**Aim:**

The aim of the study was to assess students’ perceptions of the UYDF support programme.

**Methods:**

This was an observational descriptive study. Participants were students supported by UYDF and data were collected by a questionnaire with a Likert scale to assess perceptions of various aspects of the support programme.

**Results:**

Students’ perceptions about the UYDF support programme were generally positive, with initial orientation and information sharing perceived as useful. Some respondents did not perceive value in holding discussions around English proficiency. The support required appeared to diminish with increasing years of study.

**Conclusion:**

A comprehensive, proactive compulsory support system that provides both academic and social support was perceived as useful by the UYDF students. Further research is required around aspects such as encouraging English proficiency. In future, the support programme could prioritise students in the early years of their study.

## Introduction

Staffing of rural healthcare facilities constitutes an ongoing challenge globally, with many rural areas experiencing greater shortages of staff than urban areas.^1,2,3^ International and local evidence has shown that rural-origin graduate students are more likely to provide long-term services in rural healthcare facilities than urban-origin students.^4,5^ Despite the evidence, it is of concern that South African Health Science Institutes of Higher Learning (IHL) do not currently prioritise the selection of rural-origin students, and in fact some policies (e.g. the National Bench Mark Examination) discriminate against increasing access for rural-origin students.^6^

Increasing access and throughput at IHLs for disadvantaged Black African students has been a long-standing priority in South African universities.^7^ Over the last 20 years, the number of Black African students accessing IHLs has risen from 30% in 1990 to 66% in 2010.^8^ Unfortunately, increasing access has not equated to an increasing number of Black students graduating.^9^ Although better in the health sciences,^9^ cohort studies have shown that graduation rates of Black African students at IHLs in the life sciences, mathematics and physical sciences are about 33% (which is about half that of White students), with no disaggregated data on the proportion of rural-origin students graduating.^9,10,11^

South African IHLs have responded to the challenges faced by Black students by developing various types of academic support programmes specifically designed to bridge the gap between the academic competence of the student and the requirements of the IHLs.^7,10,12^ Many of these programmes have shown success, with participating students achieving graduation more often than students who do not have access to them.^10^

Criticisms of such programmes include that they may inadvertently continue to entrench marginalisation and discrimination, as previously disadvantaged students are treated differently from other students.^7,13^ Other concerns arise around such support programmes operating from a deficiency model, being reactive and not proactive (responding to a problem and not preventing a problem) and focusing almost exclusively on academic enhancement with relative neglect of other aspects of support (such as social and psychological factors).^7,12^ ‘Extended’, ‘bridging’ and ‘additional support’ programmes, which select Black students to be trained separately from other students, may also perpetuate a perception of inferiority among students.^13^ Such a hidden curriculum may label students as weak and more likely to fail, which in turn may foster students’ resentment and non-participation.^12^

**FIGURE 1:**
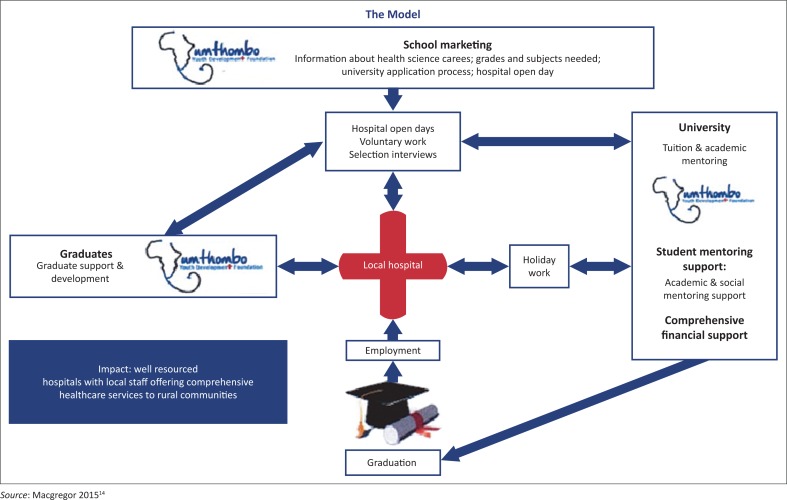
Diagrammatic representation of the UYDF model.

In response to challenges of staffing rural district hospitals, the Umthombo Youth Development Foundation (UYDF) initiated a scholarship scheme in 1999. The scholarship scheme is a partnership between UYDF and selected rural district hospitals in the province of KwaZulu-Natal (KZN) with the aim of improving healthcare delivery at these hospitals through the identification, support and training of rural-origin scholars willing to return to serve in rural areas as healthcare professionals (HCPs) after graduation. See Figure 1 for a diagrammatic depiction of the UYDF model.

Taking cognisance of the potential challenges that may be faced by Black, rural-origin students in health professions education at urban-based IHLs, UYDF provides a comprehensive, responsive, integrated and multifaceted support programme. This includes payments for fees, food, books, accommodation and a compulsory social and academic support programme. Participation in the support programme is encouraged by linking the monthly food allowance that each student receives to participation in the programme.

The support programme is proactive and includes initial orientation at the beginning of the academic year and meeting with other UYDF students, and also provides information about aspects of university life (available support structures, study skills, time management, exam techniques, dealing with distractions and the importance of passing). Students are thus forewarned of potential challenges and are provided with many opportunities to discuss their own unique challenges with other students, with a local mentor and with the UYDF mentor. The support programme thus extends beyond academic matters, as social and psychological support mechanisms are readily available.

The support programme is responsive in that students can discuss their experiences at monthly meetings with a local mentor, who is usually a lecturer working at the IHL. The students can also discuss challenges as they arise, with the UYDF mentor coordinator, either telephonically or in person. Helping students acknowledge social and academic problems, encouraging them to utilise university resources (tutors, etc.) to address problems, and holding students accountable for following through on the plans formulated are key features of the support provided by the local mentors and UYDF mentor.

In addition, students also have opportunity to meet with HCPs at hospitals close to their homes as they are required to work for four weeks each year at the hospital where they were initially selected. The UYDF management believes that key features of the support programme are the proactive nature of the programme and the individualised, ongoing, flexible, readily available, multifaceted support that alerts students to the many types of problems they may face and helps them to acknowledge and respond to these situations. Aspects of the UYDF support programme are summarised in Table 1.

**TABLE 1:** UYDF support programme.

Process	Description of activities	Key person responsible
Orientation and information sharing	Student support available at the university Drug abuse/pregnancy HIV/AIDS Examination and study techniques Potential distractions The UYDF: objectives, policies and support provided	UYDF mentor
Ongoing support Monthly contact with local mentor Biannual contact with UYDF mentor	Setting goals Support to achieve goals Monthly meetings to access academic and personal progress Review test and examination results Encourage students to identify their problems and develop a plan to address issues identified Hold students accountable to make the necessary changes Monthly report sent to UYDF mentor Discuss strategies to improve English proficiency	UYDF mentor Local mentors
Holiday work	Compulsory four weeks per year working at the district hospital where students were selected	Students UYDF mentor Local hospital staff

UYDF, Umthombo Youth Development Foundation.

Since inception in 1999, 252 rural-origin HCPs supported by the UYDF have graduated, and more than 90% of those supported by the UYDF have passed each year and progressed to the next academic year.^14^ This rate of success of student progression and graduation is set against a context in which the students were selected from one of the most disadvantaged areas of the country^15^ and the average throughput at South African IHLs.^9^

Providing support to students at IHLs is a complex and changeable intervention. In addition, it is challenging to fully describe and standardise an intervention that is intended to be organic and responsive to needs. The UYDF support programme has not been formally evaluated, and this study begins a process of evaluation by seeking the views of students on programme.

## Methods

This was an observational, descriptive study. Inclusion criteria were all UYDF students who had participated in the support programme for at least one year. A total of 201 students were supported by the UYDF in 2014, of whom 138 met the inclusion criteria.

Data were collected using a questionnaire to gather information on students’ perception of the support they had experienced. The questionnaire was designed by the primary researcher (the UYDF mentor) with input from the UYDF management team and was piloted among five local UYDF mentors and five students. Minor changes were made to the questionnaire after the pilot study and data from the pilot study were not analysed with the study data. Questions were presented as a Likert scale, with participants being asked to assess various aspects of the UYDF support programme. The scores on each aspect ranged from 1 to 5, where 1 was ‘Not useful’ and a score of 5 was ‘Extremely useful’. Aspects that were studied included perceptions of the orientation, ongoing contact with the local mentor and UYDF mentor coordinator, holiday work and participating in the annual Life Skills Imbizo.

An e-mail with the study aims, a study information sheet, a consent form and the questionnaire were sent to all eligible students, inviting them to participate anonymously in the study. Eligible students were requested to download the consent form and the questionnaire, self-complete these forms and fax or post these to the researcher, or return them to the researcher in a sealed envelope when he visited their campus. Students were specifically asked not to put their names on the questionnaire.

Data were entered into an Excel spreadsheet and analysed descriptively as mean values of the Likert scale scores with standard deviations. A consecutive one-way analysis of variance (ANOVA) was employed to assess whether there were any significant differences between various variables such as students’ year of study and perceptions about aspects of the UYDF support strategy. A *p* value of <0.05 was considered to be significant.

### Ethical consideration

Permission to conduct the study was obtained from the Humanities and Social Science Research Ethics Committee at the University of KwaZulu-Natal (UKZN) (HSS/0616/014M). Permission to collect data was also obtained from the Director of UYDF.

## Results

Of the 138 eligible students, 109 completed the questionnaire, giving a response rate of 79%. Of the participants, 55% were female, aged between 18 and 30 years, with a mean age of 21.3 years. The participants attended a variety of universities, with the majority attending the Nelson R. Mandela School of Medicine at UKZN. The universities attended are summarised in Table 2.

**TABLE 2:** Universities attended by the students that were supported.

Universities attended	No. of students	%
UKZN – Nelson R. Mandela School of Medicine	41	37.6
UKZN – Westville	29	26.6
University of Limpopo – Medunsa Campus	10	9.2
University of Pretoria	6	5.5
University of Cape Town	5	4.6
Durban University of Technology	5	4.6
Stellenbosch University	3	2.8
Nelson Mandela Metropolitan University	3	2.8
University of Zululand	3	2.8
UKZN – Pietermaritzburg	2	1.8
Rhodes University	2	1.8
**Total**	**109**	**100.0**

UKZN, University of KwaZulu-Natal.

Participants were studying a wide range of courses, with half enrolled for Medicine followed by Physiotherapy. The HCP disciplines are summarised in Table 3.

**TABLE 3:** Healthcare disciplines being studied.

Healthcare discipline	No. of students	%
Medicine	54	50.0
Physiotherapy	11	11
Pharmacy	8	7.4
Optometry	7	6.5
Radiology	7	6.5
Nursing	6	5.6
Occupational therapy	5	4.6
Audiology	4	3.7
Dietetics	3	2.8
Dental therapy	2	1.9
Dentistry	2	1.9
**Total**	**109**	**100**

Most of the participants were in their second (30%) and third year (42%) of studies, with the remainder in their fourth or fifth years.

The mean values and standard deviations of students’ perceptions of the UYDF support strategy are summarised in Table 4.

**TABLE 4:** Students’ perceptions of the UYDF support strategy.

Statements	Mean Likert score	Standard deviation
**Orientation**		
It was useful for me to set goals with the UYDF mentor at the orientation meeting	4.68	0.665
It was useful knowing what UYDF offers	4.63	0.608
It was useful to meet the UYDF mentor at the start of my studies	4.54	0.662
It was useful to meet other students supported by UYDF	4.51	0.702
The orientation given by UYDF at the start of my studies was useful	4.49	0.634
It was useful to know about student support systems	4.49	0.603
It was useful to discuss studying for examinations and to be shown how to study	4.48	0.676
It was useful to receive information on drug abuse/sexually transmitted infections/pregnancy/HIV and AIDS	4.21	0.887
It was useful to discuss potential distractions	4.07	0.861
It was useful to discuss improving English proficiency	3.57	0.963
**Mentoring**		
It was useful to have biannual contact with UYDF mentor	4.62	0.676
It was useful to have a discussion on setting high targets	4.57	0.740
It was useful for the UYDF mentor to share his experiences at university	4.47	0.717
It was useful to have a monthly meeting with my local mentor	4.18	0.945
It was useful to discuss my results with my local mentor	4.10	1.015
**Holiday work**		
Holiday work was useful	4.56	0.759
**Annual Imbizo**		
The annual Imbizo was useful	4.46	0.866

UYDF, Umthombo Youth Development Foundation.

The majority of respondents felt that it was useful to meet the UYDF mentor at the start of their studies and found the initial orientation at the start of the academic year to be useful. The participants also felt that the information given during the orientation (such as the availability of student support systems, study techniques, information on drug abuse, HIV, sexually transmitted infections and pregnancy prevention and time management) was useful. The information on speaking and listening to English as a way to improve their competency and overall success was not perceived to be as useful as other information (mean 3.57). However, there was a large standard deviation in responses (0.963), suggesting that some students found it very useful and others not at all. Participants felt that it was valuable to express their expectations and found worth in knowing about the role of the UYDF and what the UYDF offers to students.

A consecutive one-way ANOVA showed that students in the early years of study found the monthly meetings with the local mentor more useful compared to students in the later years of study.

## Discussion

The data showed that students studying a wide range of disciplines are supported by the UYDF, with a large number of different universities attended. This reflects the large number of services provided at rural district-level hospitals and the importance the hospitals attach to the training of staff to provide these services. Those 11 universities being attended also present challenges for UYDF in providing a support service that is spread across South Africa.

The current UYDF support strategy is multifaceted and focuses on social, psychological, financial and academic support, and it was encouraging to find that students’ perceptions were generally positive. Other studies have shown that succeeding at an IHL requires major social, emotional and academic adjustments, and underprepared students from disadvantaged backgrounds often experience feelings of inadequacy, alienation and isolation at an IHL.^16,17^ In addition, rural (disadvantaged) students arriving at an IHL find the university environment intimidating.^16^ They often have poor studying techniques, are unable to deal with the large volume of work at IHL level and struggle to study independently.^10^ In light of these studies, it was encouraging that students felt the UYDF mentoring was beneficial to them.

Participants found the initial orientation to be of value, and this may be because students may have never previously travelled beyond their local area into a city. The need to orientate South African HCP students into a place of learning is reflected in South African literature.^18^ This need to support students during the transition from school to an IHL is also supported in international literature.^19^

Students also perceived information sharing around issues such as HIV/AIDS, drug abuse and preventing pregnancy as useful. To an outsider this may seem strange, as it would be assumed that HCP students would already have knowledge of such issues. However, knowledge cannot be taken for granted; the literature reveals that HIV/AIDS prevention knowledge was significantly lower in South African rural school leavers compared with their urban counterparts.^20^ Similarly, the need to provide information around substance abuse for rural learners is also supported by the local literature.^21^

Students found it useful to discuss expectations early on and set high standards which they could aspire. Morales has suggested that high expectations based on the strengths, interests, hope and dreams help students tap into their intrinsic motivation and own desire for learning and personal gain.^22^ Bernard has also highlighted the importance of supportive relations that encourage students to aim high, which can make a difference in the academic life of students even when they come from dysfunctional families and poorly functioning schools.^23^

The UYDF support programme is premised on the belief that students have the potential to succeed, that any deficiencies from their schooling are challenges that can be overcome, and that they are solution-finders who are destined for success. Encouragement to participate in IHL support programmes was therefore approached not from a ‘deficiency perspective’ but rather from the perspective of one who has already successfully overcome previous challenges and who sees another problem simply as another challenge that can also be overcome.

Students reported that they found the ongoing support provided by the UYDF mentor and local mentors to be useful. The emphasis on regular contact with both the local and the UYDF mentor was perceived as important and ensured that students were held accountable to follow through on plans made to address deficiencies identified. This finding is encouraging as research indicates that Black students are less likely to access counselling services available at IHL despite facing serious problems.^24^

In addition, students found the contact with other students who were also supported by the UYDF to be useful. A peer group of like-minded students who understand the context from which they have come, and is able to provide social and academic support, has been shown to be effective in reducing students’ feelings of alienation and to assist their integration into IHLs.^25^ Emphasis beyond academic support to include social support and social engagement has been shown to be important in contributing to academic success at IHLs.^25,26,27^

Research has shown that participating in academic support systems, such as communities of learning (as offered by UYDF), increases student productivity and quality of effort that students put into learning activities to ultimately succeed at IHLs.^25,28^ Tinto reported that students who participate in communities of learning show greater engagement in social and academic activities, greater motivation, greater support and encouragement, access more university academic resources, get more support and show a greater willingness to participate in activities because they feel valued and appreciated.^25^ In addition, participation in communities of learning encourages reflection and an honest assessment of the self, a willingness to risk acknowledging deficiencies and a searching for solutions, which are important prerequisites for help-seeking behaviour.^22^ The UYDF support programme facilitates and encourages the formation of such communities of learning, and also encourages critical self-reflection to identify knowledge gaps that need to be addressed, which the UYDF students acknowledged as important.

It was interesting that students did not perceive the advice given around improving their English to be as useful as other aspects of the support programme. For many, English is a second (or third) language, which may lead to poor comprehension and reduced literacy, which in turn have been identified as important factors in student failure at IHLs.^7,16,17^ This finding requires further research as South African studies indicate that English proficiency influences academic performance and also stress the importance of enhancing language proficiency when teaching in a multicultural context.^29,30^

In addition to the on-campus support, UYDF students are expected to work for at least one month during their vacations at the hospital where they were selected. The UYDF students perceived their holiday work to be useful and this is reflected in the literature that shows career-specific experiential work as an important motivating influence for students at IHLs.^10^ Other authors have shown that experiential work during vacations makes a significant contribution to academic success, as students recognise the value of what they are learning and see the application of theory and knowledge from their IHL transferred to a real-life workplace.^10^

Academic and social support appeared to be most valued during the early years of study, and this is reflected in literature that reports that support is required to reduce thoughts of isolation experienced by HCP students in the early years of their studies.^30^ Some authors have suggested that any support strategy should assist students to develop ‘universal survival skills’ that will help them to successfully transition from being a student to becoming a graduate.^10^ That students identified some aspects of support as less important in the senior years is encouraging, as it suggests that they have adapted to the demands of tertiary education and are developing ‘survival skills’. They no longer need such intensive support, which then allows the UYDF mentor and local mentors to concentrate their efforts on students in earlier years of study.

## Limitations

Although every effort was made to ensure that responses were anonymous, students may have perceived that their responses could become known and this might have affected their responses to the questionnaire. There may have been potential bias in that participants may have responded in an overly positive manner to please the researcher. The questionnaire adopted a deductive methodology in which the researcher set the variables according to his knowledge and experience of the support programme. A more inductive method may facilitate students speaking about their own experiences, and thus data should be triangulated by further study including methods such as interviews with students and focus group discussions. The questionnaire did not permit for disaggregation and focus on specific aspects of the UYDF programme (e.g. the role of comprehensive financial support, the selection process and the student Life Skills Imbizo), which may be key in facilitating student success at IHLs. In addition, utilising the term ‘useful’ in all of the questions was a non-specific term with an interpretation on what it means that may have varied from one student to another.

## Conclusion and recommendations

UYDF-supported students hail from deep rural areas, do not speak English as a first language and may never have travelled outside their local area prior to attending an IHL. They may be more disadvantaged than their urban colleagues, as rural schools generally prepare learners poorly for tertiary education. The UYDF team can be assured that the multifaceted student support strategy was perceived as generally useful by students. The potential link between a high-quality support programme and high graduation success rates requires further study. This study reported on one specific support programme and the lessons learnt around integration, being proactive and responsive, which may be useful to other support programmes offered to disadvantaged students attending IHLs.
